# Draft Genome Sequences of Four Bacillus thermoamylovorans Strains Isolated from Milk and Acacia Gum, a Food Ingredient

**DOI:** 10.1128/genomeA.00165-15

**Published:** 2015-03-26

**Authors:** Antonina O. Krawczyk, Erwin M. Berendsen, Robyn T. Eijlander, Anne de Jong, Marjon H. J. Wells-Bennik, Oscar P. Kuipers

**Affiliations:** a Molecular Genetics, University of Groningen, Groningen, the Netherlands; b NIZO food research, Ede, the Netherlands; c Top Institute Food and Nutrition (TIFN), Wageningen, the Netherlands

## Abstract

The thermophilic bacterium Bacillus thermoamylovorans produces highly heat-resistant spores that can contaminate food products, leading to their spoilage. Here, we present the whole-genome sequences of four B. thermoamylovorans strains, isolated from milk and acacia gum.

## GENOME ANNOUNCEMENT

Bacillus thermoamylovorans is a facultative thermophilic, facultatively anaerobic, amylolytic bacterium that was isolated from palm wine and characterized first in 1995 (1). The species is being studied because of its ability to produce lactic acid (2) and a thermostable lipase (3) as well as to degrade sewage sludge (4) and plant biomass (5). The bacterium was also found to contaminate gelatin extracts (6) and has been isolated on dairy farms (7, 8). B. thermoamlyovorans forms spores (7) that are highly heat resistant; these can survive preservation treatments that are commonly used by the food industry, and upon germination and outgrowth, this can lead to food spoilage (8, 9).

Four strains of B. thermoamlyovorans, isolated from foodstuffs in which spoilage occurred, were subjected to next generation whole-genome sequencing. The isolates were cultured overnight in brain heart infusion (BHI) broth (Difco) supplemented with vitamin B_12_ at 50°C with shaking (220 rpm). After being harvested, the cell pellets were resuspended in SET buffer (75 mM NaCl, 25 mM EDTA, 20 mM Tris-HCl, pH 7.5). The cell suspensions were treated with lysozyme (2 mg/ml) and RNase (0.4 mg/ml) at 37°C for 30 min. Subsequently, the samples were incubated with proteinase K (0.5 mg/ml) and SDS (final concentration, 1%) at 55°C for 60 min. Genomic DNA was isolated from lysed cells by phenol-chloroform extraction and precipitation with isopropanol and sodium acetate (300 mM). Precipitated DNA was dissolved in TE buffer.

### Nucleotide sequence accession numbers.

The genome sequences of the four Bacillus thermoamylovorans strains have been deposited as whole-genome shotgun projects at DDBJ/EMBL/GenBank under the accession numbers listed in Table 1.

**TABLE 1:** B. thermoamylovorans sequenced strains and their sources

Strain no.^*a*^	Source	Accession no.
B4064	Acacia gum	JXLR00000000
B4065	Acacia gum	JXLS00000000
B4166	Milk	JXLT00000000
B4167	Milk	JXLU00000000

a Numbers refer to strain collections at NIZO food research and University of Groningen (Molecular Genetics).

## References

[1] Combet-Blanc Y, Ollivier B, Streicher C, Patel BK, Dwivedi PP, Pot B, Prensier G, Garcia JL 1995 *Bacillus thermoamylovorans* sp. nov., a moderately thermophilic and amylolytic bacterium. Int J Syst Bacteriol 45:9–16. doi:10.1099/00207713-45-1-9. 7857812

[2] Combet-Blanc Y, Dieng MC, Kergoat PY 1999 Effect of organic complex compounds on *Bacillus thermoamylovorans* growth and glucose fermentation. Appl Environ Microbiol 65:4582–4585. 10508092 10.1128/aem.65.10.4582-4585.1999 PMC91610

[3] Deive FJ, Álvarez MS, Morán P, Sanromán MA, Longo MA 2012 A process for extracellular thermostable lipase production by a novel *Bacillus thermoamylovorans* strain. Bioprocess Biosyst Eng 35:931–941. doi:10.1007/s00449-011-0678-9. 22237683

[4] Ivanov VN, Wang JY, Stabnikova OV, Tay ST, Tay JH 2004 Microbiological monitoring in the biodegradation of sewage sludge and food waste. J Appl Microbiol 96:641–647. doi:10.1111/j.1365-2672.2004.02182.x. 15012800

[5] Koeck DE, Wibberg D, Maus I, Winkler A, Albersmeier A, Zverlov VV, Pühler A, Schwarz WH, Liebl W, Schlüter A 2014 First draft genome sequence of the amylolytic *Bacillus thermoamylovorans* wild-type strain 1A1 isolated from a thermophilic biogas plant. J Biotechnol 192:154–155. doi:10.1016/j.jbiotec.2014.09.017. 25270021

[6] De Clerck E, Vanhoutte T, Hebb T, Geerinck J, Devos J, De Vos P 2004 Isolation, characterization, and identification of bacterial contaminants in semifinal gelatin extracts. Appl Environ Microbiol 70:3664–3672. doi:10.1128/AEM.70.6.3664-3672.2004. 15184171 PMC427776

[7] Coorevits A, Logan NA, Dinsdale AE, Halket G, Scheldeman P, Heyndrickx M, Schumann P, Van Landschoot A, De Vos P 2011 *Bacillus thermolactis* sp. nov., isolated from dairy farms, and emended description of *Bacillus thermoamylovorans*. Int J Syst Evol Microbiol 61:1954–1961. doi:10.1099/ijs.0.024240-0. 20833876

[8] Scheldeman P, Pil A, Herman L, De Vos P, Heyndrickx M 2005 Incidence and diversity of potentially highly heat-resistant spores isolated at dairy farms. Appl Environ Microbiol 71:1480–1494. doi:10.1128/AEM.71.3.1480-1494.2005. 15746351 PMC1065131

[9] Scheldeman P, Herman L, Foster S, Heyndrickx M 2006 *Bacillus sporothermodurans* and other highly heat-resistant spore formers in milk. J Appl Microbiol 101:542–555. doi:10.1111/j.1365-2672.2006.02964.x. 16907805

